# Post-stroke Arrhythmias: Performance of Brain-heart Crosstalk Networks

**DOI:** 10.2174/011573403X363465250407072854

**Published:** 2025-04-23

**Authors:** Longxiao Liu, Bangqi Wu, Jingjie Huang

**Affiliations:** 1 Tianjin University of Traditional Chinese Medicine, 88 Changling Road, Tianjin 301617, China;; 2 First Teaching Hospital of Tianjin University of Traditional Chinese Medicine, National Clinical Research Center for Chinese Medicine, Acupuncture and Moxibustion, 88 Changling Road, Tianjin 300381, China

**Keywords:** Stroke, heart disease, arrhythmia, brain-heart axis, brain damage, systemic inflammation

## Abstract

Stroke and heart disease are two of the leading causes of the global disease burden. However, modern research has gradually revealed a potential causal link between these two conditions. Most studies have focused on the direct role of arrhythmias in stroke. However, clinical evidence suggests that the incidence of arrhythmias increases after stroke in patients without a history of arrhythmia, and cardiac disease after stroke has become the second leading cause of death after stroke. This article focuses on arrhythmias after stroke and reviews brain-heart crosstalk after stroke. This article examines the potential mechanisms of brain-heart interactions after stroke, including increased catecholamines due to autonomic imbalance, gut microbial dysbiosis, immune response, and systemic inflammation. In addition, this article discusses the impact of arrhythmia on stroke severity and the role of brain injury sites in brain-heart interactions. To address these mechanisms, we propose that post-stroke arrhythmia is a type of stroke-induced disease distinct from primary arrhythmia. We aimed to identify new therapeutic targets and treatments, both pharmacological and non-pharmacological, to achieve targeted treatment and provide guidance for future clinical prevention and treatment.

## 1 INTRODUCTION

To investigate the relationship between stroke and cardiac disorders, we conducted a literature search in the PubMed database using the following terms: Heart, Arrhythmias, Cardiac, Cardiac, Stroke, Post-Stroke, Cerebral Ischemia, Cerebral Hemorrhage, Brain-Heart Axis, and Stroke-Heart Syndrome. Initially, titles and abstracts were screened to exclude irrelevant studies or those without full-text availability. The remaining articles underwent full-text evaluation, and studies investigating the mechanisms of post-stroke arrhythmias-including clinical research, animal experiments, and reviews—were ultimately included. Through the literature review, we observed that heart disease is a known risk factor for stroke; however, this relationship is not unidirectional. The brain-to-heart relationship was first described by Byer *et al.* in 1947, who reported that cerebrovascular disease leads to cardiac muscle damage and arrhythmias [1], since then the interaction between the brain and the heart has been identified in a wide range of studies, and its mechanisms have gained particular attention in modern research. Clinical findings have shown that stroke patients are highly susceptible to developing serious cardiovascular complications, known as stroke-heart syndrome (SHS) [2], and approximately 2-6% of all stroke patients die from cardiac causes within the first 3 months. Post-stroke cardiac complications are the second leading cause of post-stroke deaths [3, 4], with cardiac arrhythmias being the most common type of cardiac complication in clinical practice [2, 5]. Several studies have reported cardiac abnormalities following acute stroke. More than 60% of patients with ischemic stroke develop electrocardiogram (ECG) abnormalities [6], and 29.5% of patients develop significant arrhythmias [7], such as atrial fibrillation (AF), ventricular ectopic beats, supraventricular tachycardia, and ventricular supraventricular tachycardia [8, 9]. However, their incidence varies among different studies due to differences in stroke-related factors such as patient age, gender, type and onset of stroke, duration, medical history, and treatment [10] *etc,* (Table **1**) [9, 11-17].

This review builds upon the literature by elucidating the intricate crosstalk network between the brain and heart. It employs the example of the most prevalent clinical post-stroke arrhythmias to illustrate this network.

Poststroke arrhythmias are not clearly defined, but they are considered part of the manifestations of SHS and are new evidence of poststroke cardiac changes. The time window for their occurrence is specified as up to 30 days after a stroke. Cardiac events that occur beyond this time window are defined as possible long-term complications due to a weaker correlation with stroke [18]. Post-stroke arrhythmias may go unrecognized due to their self-limiting and transient nature [19]. Patients with cardiac symptoms after stroke have higher mortality and recurrence rates than those without cardiac symptoms [12, 20]. This raises the question of whether post-stroke arrhythmias differ from primary arrhythmias. A number of animal studies, clinical studies, and neuroimaging studies have provided increasing evidence that poststroke-induced alterations in autonomic control of the heart appear to play a critical role, but the underlying mechanisms of pathology are unclear, and therapeutic targets require further investigation [21]. This review analyzes the possible mechanisms of post-stroke arrhythmias in terms of autonomic nervous system (ANS) imbalance, inflammatory effects, and intestinal alterations. The general clinical features and potential mechanisms of post-stroke arrhythmias are systematically outlined to provide a new theoretical basis and further research direction for clinical treatment and prevention.

## 2 THE CO-MORBID BASIS OF THE BRAIN AND HEART

The heart and the brain are not independent systems but are fundamentally interconnected through the neurovascular and humoral pathways that form the brain-heart axis, and there is a certain similarity between the two in terms of their pathophysiology. In terms of anatomical structure, the vascular distributions of the heart and the brain are similar, with large arteries on the surface of the organ on the one hand, and resistance arterioles and smaller vessels penetrating deeper into the organ on the other hand [22]; at the same time, similar macrophages are present in the heart and the brain, and interact in inflammatory immunity [23]. The innervation of the heart involves the intracardiac nervous system (ICNS, also referred to as the intrinsic nervous system) and the extracardiac nervous system (ECNS) [2, 24, 25]. The ICNS comprises sympathetic and parasympathetic postganglionic efferent neurons, local circuit neurons, and afferent neurons [26, 27]. These processes are regulated by ECNS [28]. Sympathetic and parasympathetic fibers enter the heart to form synapses [29]. The intrinsic cardiac nervous system serves as a relay station, modulating heart rate and myocardial contractility through a centralized autonomic nervous (CAN) network. It forms a feedback loop with the spinal cord, brainstem, hypothalamus, and forebrain to synergistically control cardiac functions [26]. It has been shown that the left ventricle of the heart exhibits a strong correlation with the microstructure of the brain's white matter fiber tracts, which indicates that worse white matter microstructure is closely associated with poor cardiac status [30]. These findings suggest that the brain and heart have a clear basis for co-morbidity and that functional impairment of various types of heart function is commonly observed after all kinds of cerebrovascular events.

Meanwhile, results from prospective cohort studies suggest that multiple risk factors can have a common effect on the brain and heart, such as smoking, insufficient physical activity, alcohol consumption, and obesity [31, 32]. Furthermore, patients with hypertension, diabetes mellitus, and atherosclerosis may develop both neurologic and cardiovascular disorders [11, 19, 33, 34], which can affect the brain-cardiac axis. In clinical observations, arrhythmia is a common clinical feature of brain-cardiac axis imbalance [35].

## 3 MECHANISMS OF POST-STROKE ARRHYTHMOGENESIS

Cardiac pacing begins with the sinus node, which is the primary pacemaker of the heart. Other tissues, such as the atrioventricular node and the His bundle, also have intrinsic automaticity. Normal excitation originates in the sinus node and propagates in an orderly fashion from the sinus node to the atria and then to the ventricles *via* the atrioventricular (AV) node [36]. Arrhythmias are classified as bradycardic or tachycardia arrhythmias, induced by abnormalities in sinus node excitation, excitation generated in cardiac tissues other than the sinus node, or slow or blocked conduction of excitation [36-38]. Clinical studies have shown that the incidence of post-stroke arrhythmia is highest in the first 24 hours and decreases over time in the first 3 days, suggesting that compensatory neuroplastic changes occur after acute brain injury [11, 39], which suggests that the clinical manifestations of post-stroke arrhythmia may result from structural or functional brain damage caused by the stroke. The main pathway of post-stroke arrhythmia is widely recognized to involve an autonomic imbalance that allows vagal and sympathetic regulation of cardiac arrhythmia through the intrinsic cardiac ganglionic plexus [28]. In addition, this paper proposes mechanisms of persistent inflammation, brain-gut-heart pathways, and feedback from cardiac damage to describe the mechanisms of post-stroke arrhythmia.

### 3.1 Autonomic Nervous System Imbalance

Several studies have identified reduced heart rate variability (HRV) in patients after acute stroke [40], suggesting that impaired cardiovascular autonomic reflexes are common after acute stroke [4, 21]. This is mainly due to the mechanism of damage to the central structures controlling the ANS, which leads to sympathetic amplification or parasympathetic inhibition of the ANS [32]. This deregulation promotes electrophysiological as well as structural alterations of cardiomyocytes, which can lead to a series of clinical alterations such as ischemic ECG, arrhythmias, and impaired cardiac function.

### 3.2 Stroke-associated Lesion Sites

There is no single arrhythmogenic center in the brain; there is an intracerebral spatial network distribution specific for ANS function, and stroke localization may have different effects on the ANS, further contributing to different degrees of cardiac injury and dysfunction [41]. Through direct measurements of brain regions associated with the ANS in humans, Beissner *et al.* identified several brain regions associated with autonomic control-related brain regions, such as the ventral medial prefrontal cortex, anterior tonsillar cortex, and insula [42]. Collectively, several studies have shown that lesions in the insula, frontal cortex, parietal cortex, cingulate cortex, amygdala, hypothalamus, and brainstem are significantly associated with the development of arrhythmias [32, 39, 43].

#### 3.2.1 Insula

The insula is hidden beneath the frontal, parietal, and temporal lobes and is often considered an important part of cardiac regulation [44], while the insular cortex itself has extensive connections with other brain regions, such as the amygdala, hypothalamus, and anterior cingulate cortex, which together integrate autonomic regulation [28]. Clinically, patients with insula or middle cerebral artery damage present with ECG changes, arrhythmias, and sudden death [45]. It has been demonstrated that when right insula damage occurs, there is an increased risk of tachyarrhythmia, prolonged QTc intervals, and left bundle conduction block [46]. At the same time, manifestations associated with cardiomyocyte injury have shown some correlation with insula injury [47], Ay *et al.* [48] reported that approximately 88% of stroke patients with right hemispheric insular cortex damage had elevated serum cardiac troponin T levels associated with myocardial injury in the weeks after stroke. Peter Sörös *et al.* [4] showed that lesions of the insula cortex, which may alter the balance of sympathetic and parasympathetic tone and increase plasma catecholamine concentrations, lead to myocardial injury and increase the incidence of heart disease. Neuroimaging studies have also revealed patterns of activation related to cardiovascular and autonomic nerves. Krause *et al.* observed by magnetic resonance imaging that the dorsal anterior insula cortex plays an important role in the parasympathetic control of cardiac and autonomic function. Acute vascular injury in this subregion of the insula may lead to autonomic imbalance and increased sympathetic nervous system function, resulting in myocardial injury [47, 49].

#### 3.2.2 Frontal Lobe

Rydén *et al.* reported a link between lesions in the frontal region and the development of AF [50], and the prefrontal cortex appears to be an important site of influence on the cardiovascular system [51]. Several studies have found that the frontal lobe has connections with the amygdala and insula, which together influence autonomic function [28, 52], and that it can control sinoatrial node excitation through a bidirectional pathway to the parasympathetic nervous system [52]. Damage to the prefrontal cortex causes an imbalance between vagal and sympathetic responses, so lesions in the frontal lobe are often associated with the development of arrhythmias, but there are hemispheric variations that need to be further investigated clinically [53].

#### 3.2.3 Parietal Lobe

Studies have shown that bilateral subparietal lobules may also be part of the central autonomic function network [54], and infarctions involving the parietal lobes appear to be associated with a particularly increased risk of cardiac death or myocardial infarction [55, 56]. There is a functional connection between the parietal cortex and the cardiac ANS [57]; therefore, infarctions in the parietal cortex may lead to autonomic dysfunction, including sympathetic hyperexcitability and impaired vagal function. This dysfunction may further lead to arrhythmias and sudden death [58].

#### 3.2.4 Amygdala

The amygdala is one of the integrating centers involved in the activity of the ANS and cardiovascular function [59]. In animal studies, stimulation of the posterior central and medial nuclei of the amygdala elicits a tachycardic response, whereas a bradycardic response is elicited by the anterior portion of the amygdala [60]. The study showed a 1.6-fold increase in the risk of cardiovascular events for each mean increase in amygdala signaling. Increased amygdala activity can cause arterial inflammation by upregulating bone marrow activity, and upregulation of hematopoietic tissue activity is associated with increased atherosclerotic inflammation [61]. Additionally, the amygdala has extensive network connections with the insula [44], and damage to the amygdala can increase sympathetic output, which can induce arrhythmias.

#### 3.2.5 Hypothalamus

The hypothalamus controls autonomic input to the heart primarily through inputs from the paraventricular nucleus, the dorsomedial nucleus of the hypothalamus, and the lateral hypothalamic area [62]. The paraventricular nucleus can project directly to the ventral-lateral aspect of the medulla oblongata cephalad and then *via* the sympathetic outflow from the heart, and stimulation of the hypothalamus to activate the sympathetic output can induce electrocardiographic abnormalities and cardiac arrhythmias [63]. Rogers *et al.* reported that stimulation of the hypothalamus in cats may produce electrocardiographic T-wave changes, increase sympathetic flow to the heart, and significantly lower the ventricular threshold of electrical sensation, inducing the onset of cardiac arrhythmias [64]. In addition, there are pathways connecting the insula cortex and the lateral hypothalamic area, and glutamate may be a neurotransmitter at this site, with the hypothalamus able to mediate the sympathetic effects of insula cortical stimulation [65].

#### 3.2.6 Cingulate Cortex

The anterior cingulate cortex has close projections to brain regions, including the amygdala, hypothalamus, and gray matter surrounding the brainstem aqueduct [66]. It has been shown that both the anterior insula and the anterior cingulate cortex contain the same population of neurons, and it is thought that these neurons form connections between these regions [67, 68]; neuroimaging and clinical evidence also suggest that the dorsal cingulate cortex supports the autonomic state that produces associated cardiovascular arousal [69]. Cingulate cortex activation was significantly correlated with high-frequency HRV spikes, suggesting a role in autonomic regulation [70]. The prefrontal cortex, amygdala, cingulate cortex, and hypothalamus are similar to the components of the limbic system [71], which are closely related to the generation of emotions in humans, and some brain regions of the limbic system are connected to the ANS so that the influence of emotional factors on the development of post-stroke arrhythmia can be considered indirectly.

#### 3.2.7 Brainstem

The sympathetic network in the brainstem plays a key role in the neural control of the cardiovascular system, and most neurotransmitter impulses that regulate cardiovascular activity are transmitted through the medulla oblongata, which is, therefore, considered to be a major cardiac autonomic center. Sympathetic nerve activity is dependent on the caudal one-third of the pontine bridge and the rostral two-thirds of the medulla oblongata [72], and the ventrolateral aspect of the medulla oblongata cephalad is considered to be an important presence of autonomic centers [73]. Autonomic downstream conduction pathways can pass directly through the pontine-bridge-medulla oblongata junction or indirectly through the periaqueductal gray (PAG) matter, and it has been found that activation of certain areas of the PAG can lead to physiological responses such as an increase in heart rate and a rise in blood pressure. The frontal lobe, insula, and amygdala can project to the periaqueductal gray matter, and the PAG also projects to the deeper layers of the thalamus, hypothalamus, brainstem, and spinal cord, which are involved in regulating cardiovascular function [74-76].

In summary, the central ANS is a complex brain network that regulates cardiovascular function by modulating sympathetic vagal outflow to the heart, where autonomic afferent nerves can transmit information to the hypothalamus, amygdala, and other cortical areas and project to, for example, the insula and prefrontal cortex, which in turn passes through the hypothalamus, medulla oblongata, or periductal gray matter [28, 77]. The networked nature of the central ANS plays a crucial role in the development of post-stroke arrhythmia, and the structure of the central ANS network may be damaged or altered, leading to the development of cardiac arrhythmia.

### 3.3 Increased Catecholamine Secretion and Calcium Ion Imbalance

**Fig. (1):**
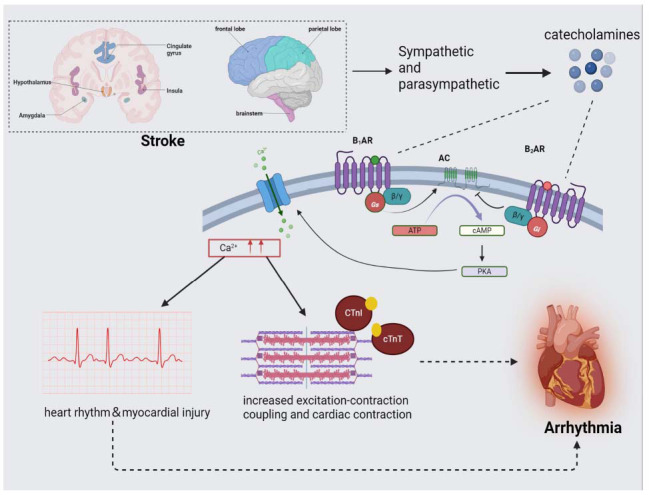
Lesions in specific brain regions can cause an imbalance of autonomic nerves, which in turn leads to an increase in catecholamines, which act on β-receptors on cardiomyocytes, activate adenylyl cyclase (AC), and increase cyclic adenosine monophosphate (cAMP) within the cytoplasm. cAMP prolongs the opening of protein-phosphorylated calcium channels through the activation of protein kinase A (PKA), which increases the amount of calcium ions in cardiac myocytes. An increase in the content of calcium ions in cardiomyocytes can affect the rhythmic conduction of cardiomyocytes on the one hand and stimulate the interaction of actin and myosin at the same time so that cardiomyocytes are in a state of continuous contraction, which damages cardiomyocytes and causes cardiac arrhythmia. **Abbreviations:** AC: adenylate cyclase; cAMP: cyclic adenosine monophosphate; PKA: protein kinase A; cTnT: cardiac troponin T; cTnI: cardiac troponin I.

Excessive sympathetic stimulation after stroke leads to increased secretion of adrenomedullary catecholamines [21, 32], which at the cellular level can have direct toxic effects on the myocardium by inducing an increase in relative myocardial ischemia [78]; in addition, the surge in catecholamines can cause remodeling changes in cardiomyocytes, such as hypertrophy and fibrosis [79, 80], which increase the instability of myocardial electrical activity, and play an important role in post-stroke arrhythmia. Second, sympathetic excitation results in the secretion of epinephrine, which acts on β-adrenergic receptors in cardiomyocytes to produce cyclic adenosine monophosphate (cAMP), which opens calcium channels, leading to an increase in the intracellular calcium concentration and an increase in the duration of the plateau phase of the cardiac action potential [28]. It also stimulates actin-myosin interactions that invalidate muscle relaxation, which leads to a state of overcontraction of cardiomyocytes and affects the electrical stability of the myocardium, including enhanced autoregulation of the sinoatrial node, excitability of the Hirshhorn-Purkinje system, early afterdepolarization, and conduction block [28, 77, 81]. This lesion is mainly located in the subendocardium and may involve the cardiac conduction system, increasing the risk of arrhythmias [82]. At the same time, increased intracellular Ca^2+^ promotes pulmonary vein cardiomyocytes, which are an important source of ectopic beats that can trigger paroxysmal AF and ectopic atrial tachycardia [83], which may play an important role in the development of arrhythmias (Fig. **1**).

## 4 BRAIN-AUTONOMIC-GUT-CARDIAC AXIS

Bidirectional signaling pathways exist between the brain and the gut and are interconnected through the ANS, the hypothalamus-pituitary-adrenal (HPA) axis, and the enteric nervous system [84]. Brain injury has been found to have a significant impact on the composition of the microbiota [85], including a reduction in the species diversity of the microbiota and an overgrowth of intestinal bacteria as well as a preferential expansion of the phylum Mycobacterium [86]; in particular, some beneficial commensals are reduced in patients and some opportunistic pathogens are increased, and this ecological imbalance correlates with the severity of brain injury [87]. Poststroke autonomic imbalance modulates the gut in many ways through sympathetic and parasympathetic nerves that synapse directly with the gut [88]. Vikramjeet *et al.* reported that extensive poststroke elevated blood catecholamine levels, which leads to the development of intestinal paralysis, and that this mediated intestinal paralysis is a potential cause of poststroke microbiota dysbiosis [86]. Overall, stroke in patients leads to a series of gastrointestinal dysfunctions, including decreased intestinal motility, increased intestinal permeability, and dysbiosis of the intestinal flora [89, 90], which mediate the development of post-stroke arrhythmia by affecting bacterial metabolites, inflammatory factors, and immune cells in the host's intestinal tract through the nerves, bloodstream, and direct action on cardiac muscle tissue.

### 4.1 Intestinal Bacteria and their Metabolites

Increased intestinal permeability after stroke may release bacteria and their metabolites into the circulation [90, 91], and trimethylamine oxide (TMAO) is the most widely studied microbial metabolite involved in the pathogenesis of cardiovascular disease [92]. Gut bacteria produce trimethylamine (TMA) from food, and then TMA enters the liver for oxidation to produce trimethylamine oxide (TMAO) [93]. Reduced expression of mucins and tight junction proteins in the gut after acute stroke leads to increased permeability of the gut barrier [90], which increases the amount of TMAO that enters the circulation, ultimately causing elevated levels of TMAO after stroke [89]. Furthermore, there is a robust correlation between TMAO and the prevalence of cardiovascular disease, particularly the occurrence of arrhythmias. The underlying mechanisms that contribute to this association involve several key factors. TMAO has been observed to enhance vascular endothelial cell dysfunction and inflammatory injury, promote platelet activation and thrombosis, and induce dyslipidemia. These effects collectively aim to foster the development of atherosclerosis and escalate the risk of cardiac arrhythmias [94-97]. In addition, TMAO instigates the cardiac autonomic nervous system, precipitating the concomitant arrhythmogenesis [98]. A study by Cheng *et al.* [99] found that TMAO-treated cardiomyocytes showed a series of electrophysiologic changes such as an imbalance of calcium ions, an increase in the L-type calcium current, and an increase in the potassium current, which increased the susceptibility to arrhythmia. TMAO has been demonstrated to induce cardiac fibrosis and remodeling of cardiac structure through the process of inflammation. The presence of fibrotic tissues has been shown to have a deleterious effect on the electrical conduction system of the heart, increasing the probability of the occurrence of arrhythmias [100]. Several clinical trials have identified TMAO as a marker and predictor of cardiovascular disease [85, 101]. In addition to TMAO, bacterial metabolites such as indolephenol sulfate, lipopolysaccharide, short-chain fatty acids, and bile acids are associated with the development of cardiac arrhythmia and are clinically important risk factors for cardiovascular disease [89, 102]; however, the mechanism by which they cause the development of post-stroke arrhythmia requires further experimental studies.

### 4.2 Immune Response after Intestinal Disorders

Approximately 70% of immune cells reside in the gastrointestinal (GI) tract [89], suggesting that the GI tract plays an important role in the systemic immune system, and recent evidence suggests that the gut microbiota plays a key role in autoimmune diseases by regulating immune homeostasis. On the one hand, increased intestinal permeability after stroke may release bacteria and their metabolites into the circulation [90, 91], thereby triggering a systemic inflammatory response; on the other hand, acute brain injury induces dysregulation of the microbiota, which can lead to alterations in T-cell homeostasis as well as their migration to ischemic areas of the brain [103]. Singh *et al.* transplanted the microbiota after ischemic stroke into mice, and the recipient showed a massive increase in the expression of interferon-γ (IFN-γ) and interleukin 17 (IL-17) cytokines, both of which represent the polarization of T helper 1 (Th1) and T helper 17(Th17) cells [86]. In parallel with systemic immune system activation, intestinal bacteria also increase the transfer of T cells from the gut to the brain, and it has been found that T cells play a decisive role in secondary neuroinflammation after cerebral ischemia; and that the intestinal microbiota is a major regulator of T-cell homeostasis [89], exacerbating local neuroinflammation by increasing the secretion of inflammatory factors, such as IL-17 and IFN-γ [86, 103]. Further exacerbation of neuroinflammation will increase the occurrence of arrhythmias, which is discussed in detail in the inflammation section Fig. (**2**).

## 5 INFLAMMATORY RESPONSE AFTER STROKE

There is growing evidence that systemic inflammation is promoted after stroke, and epidemiology suggests a strong positive correlation between the levels of inflammatory markers and the risk of cardiovascular events [104]. Neutrophil, lymphocyte, and macrophage infiltration are much greater in the myocardium of patients who die after stroke [105], and chronic inflammation and apoptosis are found in the cerebellum and heart after 6 months of transient total cerebral ischemia induction in nonhuman primates [106], suggesting a direct link between stroke and local inflammation in the heart.

### 5.1 Neuroinflammation after Stroke

Localized inflammation in the brain begins after vascular occlusion, and local ischemia can lead directly to endothelial cell damage through the release of reactive oxygen species (ROS) [107], which in turn increases the permeability of the blood‒brain barrier (BBB). Moreover, brain injury leads to the release of damage-associated molecular patterns (DAMPs), which can activate various types of immune cells, such as microglia and astrocytes. The activation of these immune cells leads to an increase in the production of pro-inflammatory cytokines (*e.g.*, IL-6, IL-1β, and TNF-α), and the large number of inflammatory factors produced can aggravate damage to nerve cells and the BBB [108-110]. Mast cells in the brain, which reside in the cerebral microvasculature at the early stage of ischemia, release cytoplasmic particles containing a large number of vasoactive mediators, which act on the basement membrane and are capable of inducing BBB damage [111]. Border-related macrophages induced by ischemia rapidly affect leukocyte chemotaxis and BBB integrity and contribute to neurological impairment in the acute post-stroke period [112].

The BBB consists of endothelial cells, astrocyte end-feet, pericytes, and thick basement membranes and is the core of the neurovascular unit [113]. Following blood–brain barrier injury, the release of DAMPs and various cytokines and chemokines into the circulation can attract immune cells such as peripheral macrophages and neutrophils to infiltrate the lesion area. Neutrophils are among the first immune cells to be recruited to the injury site and can release proteases, perforin, and inflammatory IL-1β [91]. Neutrophils have also been shown to cause BBB disruption [114]. BBB disruption promotes the entry of inflammatory factors into the brain, and inflammation increases BBB permeability, which maintains a vicious cycle. At the same time, DAMP and cytokines entering the circulation induce an immune response in lymphoid organs that can trigger systemic inflammation [91, 115].

### 5.2 Cardiac Effects of Systemic Inflammation Induced after Stroke

According to a recent analysis, the occurrence of systemic inflammation has an impact on cardiac arrhythmias [116]. Animal experiments have shown that the infiltration of proinflammatory macrophages into the heart and activation of the NOD-like receptor thermal protein domain associated protein 3 (NLRP 3) inflammasome pathway are key events that may lead to cardiac dysfunction after stroke in mice [117], and the mechanisms of inflammation in cardiac arrhythmia are mainly electrical and structural.

#### 5.2.1 Electrical Remodeling

Inflammation at the local level can directly lead to changes in the electrophysiological properties of cardiomyocytes, such as prolongation of the effective response period and prolongation of the monophasic action potential, which can lead to the development of cardiac arrhythmias. In addition, inflammatory stimuli produce cytokines, such as TNF-α [118, 119], IL-6, and IL-1 [120], which can alter cardiac electrical pathways, including hyperactivation of sodium channels, calcium ion abnormalities, and prolonged action potential duration, which affects the electrical characteristics of the atria and leads to increased heterogeneity of electrical conduction in the atria [120, 121]. The activation of the NLRP3 inflammasome cardiomyocytes can lead to alterations in cardiac electrophysiologic properties, such as a shortened effective refractory period and an imbalance in calcium homeostasis, which can trigger arrhythmias [120].

#### 5.2.2 Structure Reconstruction

**Fig. (3):**
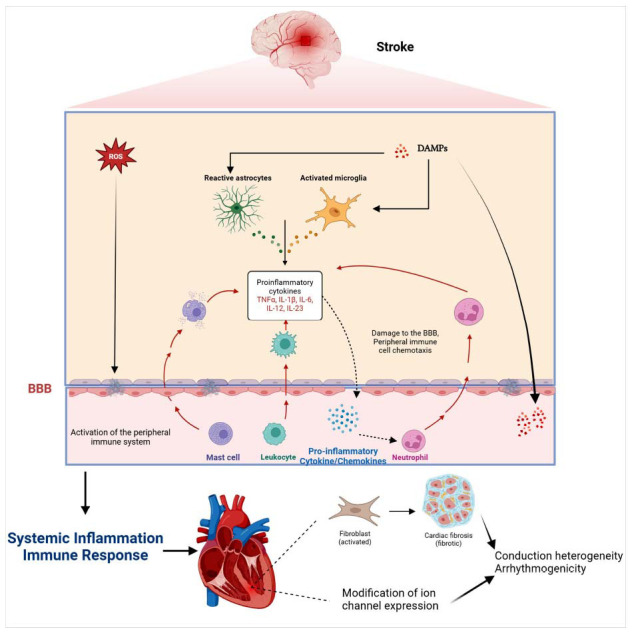
Post-stroke causes the release of local damage-associated molecular patterns (DAMPs) and reactive oxygen species (ROS) in the brain. DAMPs can activate various types of immune cells, causing an increase in pro-inflammatory cytokines and chemokines, which, together with ROS, disrupt the blood‒brain barrier (BBB) to allow their entry into the body circulation to cause the chemotaxis of local immune cells and the development of systemic inflammation. In the presence of systemic inflammation, an increase in cytokines can increase conduction heterogeneity through electrical and structural remodeling, creating an arrhythmogenic substrate. **Abbreviations:** DAMPs: damage-associated molecular patterns; ROS: reactive oxygen species; BBB: blood-brain barrier; IL-1β: interleukin-1β; TNF-α: tumor necrosis factor α; IL-6: interleukin 6; IL-12: interleukin 12; IL-23: interleukin 23.

Inflammation can lead to atrial remodeling through activation of the transforming growth factor-β (TGF-β) signaling pathway and myofibroblasts, and the cytokine interleukin-1β (IL-1β) also contributes to the transition from fibroblasts to myofibroblasts, which play important roles in cardiac fibrosis [108]. Studies have confirmed that proinflammatory cytokines can alter the sympathetic output of the HPA axis, which further drives excessive catecholamine release and exacerbates the onset of myocardial remodeling [122]. In summary, inflammation can lead to structural changes in cardiomyocytes [37], such as fibrosis, scar formation, myocardial hypertrophy, and atrophy. These changes can increase the risk of arrhythmias such as ectopic pacing activity and localized slowing of regional conduction (Fig. **3**) [120, 123].

## 6 IMPACT OF THE HEART ON BRAIN AREAS

**Fig. (4):**
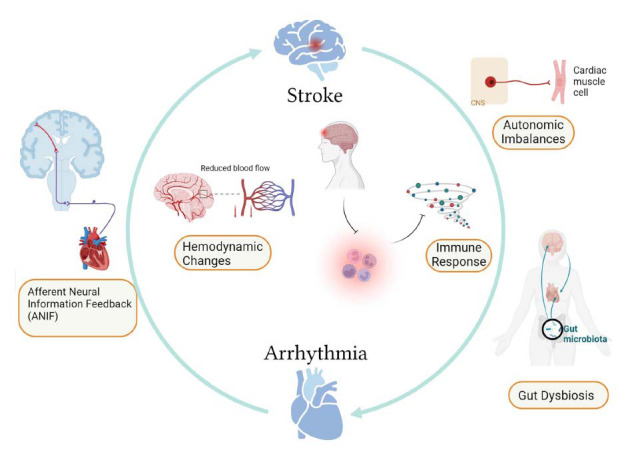
After stroke the brain can influence the heart through autonomic pathways, inflammatory responses, and intestinal disturbances, thereby increasing the risk of arrhythmia, and after arrhythmia occurs, brain lesions are further damaged due to feedback to the cerebral blood supply and cardiac afferent nerves, resulting in brain-heart crosstalk after stroke.

Cardiac arrhythmias can increase the risk of subsequent cerebral and systemic thromboembolism. In addition, hippocampal and frontal lobe damage can be observed in animal models of heart failure [124], which indicates that abnormal cardiac electrical activity in the heart may, in turn, have a direct or indirect effect on the brain. First, afferent nerves from the heart reach the nucleus tractus solitaries, where abnormal signals can be transmitted to the cerebral cortex [125], causing neuronal changes that stimulate cardiac reflexes (the so-called “cardio-cardiac reflex”). Second, following a stroke, cerebral autoregulation is impaired, so cerebral blood flow is directly dependent on cardiac function [126], in which case a reduction in cardiac output due to arrhythmia can cause further brain damage (Fig. **4**) [127].

## 7 OUTLOOK - FOR CLINICAL THERAPEUTIC USE AND PREVENTIVE GUIDANCE

According to the accumulation of clinical evidence, there is a causal relationship between brain injury and cardiac dysfunction, and it is important to analyze the clinical characteristics and mechanisms of post-stroke arrhythmia, which is a different type of disease from primary arrhythmia [128]. A study revealed a higher risk of poststroke arrhythmias in patients with cardiogenic embolic stroke [129]. The study also indicated that brain damage due to cardiogenic stroke may further affect the autonomic nervous system of the heart, inducing arrhythmias. These arrhythmias may be more severe during the poststroke period due to their complexity and potentially serious clinical consequences. However, the primary clinical concern remains the cardiac problem after stroke [122]. Future research should focus on the diagnosis and identification of post-stroke arrhythmia and clinical treatment and prevention, as well as whether there are special therapeutic drugs and methods for post-stroke arrhythmia. The main mechanisms of arrhythmia, namely, sympathetic and parasympathetic imbalance, inflammation, and intestinal microorganisms, could be promising targets for clinical research [21, 130].

### 7.1 New Paths for Preventive Monitoring

The majority of post-stroke patients remain undetectable for the development of post-stroke paroxysmal arrhythmias (usually asymptomatic), but the development of post-stroke arrhythmias has an impact on the rate of stroke recurrence [131], and the majority of stroke guidelines recommend myocardial enzyme profiling, routine electrocardiography, hourly ambulatory electrocardiography, and echocardiography for the assessment of the post-stroke arrhythmia [32]. Among these methods, power spectral analysis of HRV has been widely used [132], and HRV can specifically indicate sympathetic tone to clarify the presence of autonomic imbalances and the potential for arrhythmia development. Clinical and experimental data suggest that there is hemispheric laterality in the control of post-stroke arrhythmia [49] and that lesions in the right hemisphere are more strongly associated with the development of severe arrhythmias [39, 133], with the most definitive study of laterality being in the insula [44, 134]. The majority of sudden deaths due to severe arrhythmias are due to right insula lesions [135], and clinical monitoring of patients with infarcts in this area should be intensified.

## 8 EXPLORATION OF NEW THERAPEUTIC TARGETS

### 8.1 Pharmacological Treatment

For primary arrhythmias in the clinic, pharmacological treatments often include sodium channel blockers, β-receptor blockers, Ca^2+^ channel blockers, *etc*., which still have many side effects [36], and their therapeutic targets are focused on downstream cardiomyocytes. There are no clear guidelines for the treatment of post-stroke arrhythmia, so the same drugs are often used in clinical practice. Mild cardiac arrhythmias can be successfully treated with these drugs [11], but the therapeutic effects on severe and recurrent post-stroke arrhythmias remain to be investigated. Previous studies have shown that β-receptor blockers have a certain theoretical basis and research prospects for the treatment of post-stroke arrhythmia [2]. By blocking β-adrenergic receptors, they can prevent sympathetic over-activation and reduce excitability, and at the same time, they can prevent the increase in calcium ions and myocardial fibrosis after the catecholamine surge. Clinical animal studies have also shown that the administration of drugs such as propranolol can partially prevent post-stroke arrhythmia and further cardiac damage [136, 137] and has a certain cardioprotective effect [138], which can reduce the mortality caused by cardiac syndrome after stroke [139].

In addition, the TNF-α, IL-1b, IL-6, and IL-10 cytokines are particularly promising potential therapeutic targets for the inflammatory response to cerebral ischemia following post-stroke arrhythmia according to the mechanism of the post-stroke arrhythmia [140], and interleukin-1 receptor antagonist (IL-1Ra) has been the most extensively studied cytokine. Simats *et al.* reported that injections of IL-1Ra were effective at reducing the peripheral inflammatory response to acute stroke [141]. Craig *et al.* reported that subcutaneous injection of IL-1Ra significantly reduced plasma IL-6 and plasma C-reactive protein levels [142], and Jesus *et al.* reported that administration of IL-1Ra improved prognosis and facilitated neurogenesis after cerebral ischemia [143].

In the clinical setting, the prevention of post-stroke cardiovascular disease through the manipulation of gut bacteria in the intestinal tract has become a hot research topic. Mirian *et al.* administered Kefir probiotic bacteria to rats for a long period and found that it could improve cardiac contractility by modulating calcium-handling proteins on cardiac cells and that dopamine levels were reduced in the brainstem of the rats, which resulted in the suppression of sympathetic excitation of efferent from the brainstem [144]. Probiotic preparations specifically tailored for this purpose could form the basis of a therapeutic strategy for post-stroke arrhythmia [85]. By improving the structure of the intestinal microbial community and protecting the integrity of the intestinal barrier, the production of intestinal bacterial metabolites in the body, especially TMAO, can be reduced, thereby reducing the risk of systemic diseases [145-147].

Depending on the patient's specific clinical symptoms, we can also use medications such as managing comorbidities by controlling blood pressure, maintaining normal blood glucose levels, and using lipid-lowering medications to balance electrolyte disturbances, which play a very important role in preventing recurrent strokes as well as preventing post-stroke arrhythmia and sudden death after stroke. For example, statin use after stroke is associated with a reduced risk of cardiovascular events, and a case‒control study showed that preemptive short-term statin management reduced the risk of postoperative AF in patients undergoing cardiac surgery [148]. This may be due to the anti-inflammatory and antioxidant properties of statins, which play a cardioprotective role by reducing myocardial damage through improved cerebral blood flow and the release of inflammatory mediators localized to the trauma area [149].

### 8.2 Non-pharmacological Treatments

Recent studies have demonstrated that catheter ablation for the treatment of arrhythmias has a favorable long-term safety profile and excellent durability [35]. However, it also has a significant recurrence rate in clinical use, indicating a need for further exploration for the treatment of arrhythmias. Previous studies have shown that methods for attenuating sympathetic nerve activity, such as stellate ganglion ablation and renal sympathetic denervation, have a positive effect on myocardial electrophysiologic changes and can serve to control arrhythmogenesis [150, 151]. Mechanistically, the stellate ganglion (SG) is an important pathway between the sympathetic nervous system and the ANS of the heart. Wang *et al.* found that ablation of the left stellate ganglion (LSG) could inhibit the occurrence of ventricular post-stroke arrhythmia by down-regulating plasma levels of catecholamines and preventing macrophage activation in the myocardium in an animal study [152]. Renal sympathetic denervation reduces sympathetic tone in the heart while decreasing renal norepinephrine secretion by 47% and attenuating the activity of the renin-angiotensin-aldosterone system [153].

Palma *et al.* proposed that sympathetic nerve activity is pro-arrhythmic and that vagal activity is anti-arrhythmic in the ventricle [28]; in the ventricle, vagal stimulation prolongs the duration of action potentials and the effective refractory period of cardiomyocytes. Low-level vagal nerve stimulation (LL-VNS) has been shown to improve cardiac function in several ways. First, LL-VNS activates central vagal projections in the human brain, leading to a decrease in sympathetic output. Second, LL-VNS also reduces the release of inflammatory factors, thereby reducing the inflammatory response. Finally, LL-VNS can reverse the electrophysiological remodeling of cardiomyocytes in terms of decreased heart rate and increased expression of small conductance calcium-activated potassium channels [154-156]. Hadaya *et al.* reported that low-intensity acute VNS decreased the likelihood of developing arrhythmias, while high-intensity VNS initially caused tachycardia, followed by bradycardia [157]. Stavros *et al.* conducted animal studies and found that low-level tragus stimulation (LLTS) through completely noninvasive percutaneous stimulation could similarly reduce the incidence of arrhythmias [158]. Additionally, it may be worthwhile to explore noninvasive or minimally invasive methods of autonomic modulation in humans as a potential treatment for post-stroke arrhythmias. This finding has significant implications for the development of VNS stimulation programs.

Recent studies have shown that microglia may play an important role in cardiovascular function by releasing a variety of substances such as cytokines, chemokines, and growth factors [159, 160]. Light-emitting diode (LED) therapy has been shown to attenuate the neuroinflammatory response by inhibiting microglial activation; moreover, LED therapy reduces local cell death and attenuates the NLRP3 inflammasome while downregulating the pro-inflammatory cytokines IL-1β and IL-18 in the ischemic brain [161] and increasing the levels of beneficial neuroinflammatory markers in the brain [162]. These findings suggest that our LED therapy could be a research direction for controlling the occurrence of post-stroke arrhythmia.

For the treatment of post-stroke arrhythmia, we need to find new therapeutic targets that are different from those for primary arrhythmia in order to achieve targeted treatment. Although the above-proposed methods to achieve post-stroke arrhythmia suppression are encouraging, especially for vagus nerve stimulation methods, both auricular stimulation and acupuncture stimulation have great clinical research and application value, and further in-depth investigations of their effectiveness and safety are needed in the future.

## CONCLUSION

Although our understanding of brain-heart crosstalk after stroke has advanced considerably, the roles of sympathetic control, gut ecological dysregulation, and inflammatory responses remain to be investigated. More clinical trials are needed to demonstrate and discover the optimal therapeutic management of patients with post-stroke arrhythmia to improve cardiac function and minimize secondary brain damage. In conclusion, the occurrence of post-stroke arrhythmia remains a serious problem that requires attention, and although specific interventions are necessary to prevent and treat cardiac complications after stroke, data to guide the management of these complications are lacking.

## Figures and Tables

**Fig. (2):**
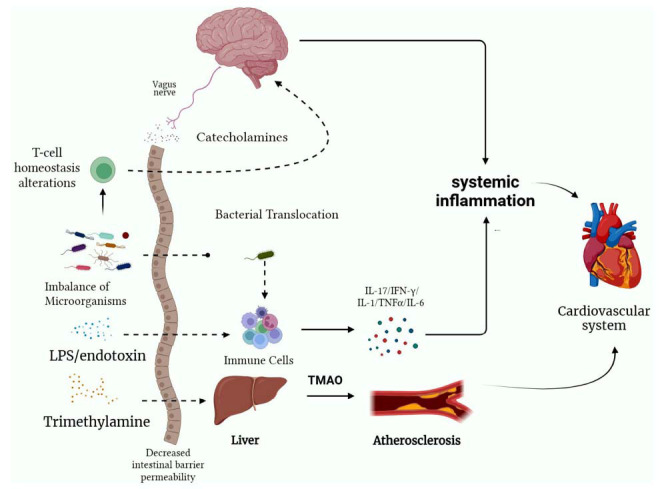
Stroke can disrupt intestinal microbes and metabolites through enteric neuromodulation and increase intestinal barrier permeability, allowing bacteria and endotoxins to translocate into the circulation. The immune response in the gut after stroke can also lead to alterations in T-cell homeostasis and T-cell migration to ischemic areas of the brain, both of which induce systemic inflammatory responses and pro-inflammatory cytokine production. Cytokine production can trigger inflammation, fibrosis, microvascular and myocardial dysfunction. The microbial metabolite trimethylamine (TMA) can be oxidized by the liver to develop trimethylamine oxide (TMAO), which promotes the production of atherosclerosis and increases the incidence of cardiac arrhythmias through multiple pathways. **Abbreviations:** IL-17: interleukin 17; IFN-γ: interferon-γ; TNF-α: tumor necrosis factor α; IL-1: interleukin 1; IL-6: interleukin 6; TMA: trimethylamine; TMAO: trimethylamine oxide.

**Table 1:** Epidemiology of post-stroke arrhythmias.

**Author**	**Brain Lesion**	**n**	**Recording Method**	**Duration Time**	**Classification of Arrhythmia**	**Incidence Rate**
Kallmünzer *et al.* [11]	Ischemic stroke Intracerebral hemorrhage	501	Continuous cardiac monitorinG	72 h	Tachycardia arrhythmia	19.20%
					Bradycardic arrhythmia	8.40%
Buckley *et al.* [12]	Ischemic stroke	365383	Continuous cardiac monitoring	4 week	Atrial fibrillation/flutter	8.80%
					Severe ventricular arrhythmias	1.20%
Carrarini *et al.* [9]	Ischemic stroke	120	Holter ECG	7 day	Bradycardia	6%
					Atrial fibrillation	4%
					SVEB	94%
					VEB	88%
					SVRs	54%
					Supraventricular tachycardia	20%
Pothineni *et al.* [13]	Ischemic stroke Intracerebral hemorrhage TIA	28	Cardiac rhythm monitoring device	14 day	Pause or atrioventricular block	7.14%
					Atrial fibrillation	7.14%
					Brief atrial tachycardia	42.86%
					NSVT	7.14%
					Premature ventricular contractions	10.71%
Muggeridge *et al.* [14]	Ischemic stroke Intracerebral hemorrhage TIA	100	R-TEST monitoring devices	7 day	Atrial fibrillation	8%
Poh *et al.* [15]	Ischemic stroke	709	Continuous cardiac monitoring	24 h	Atrial fibrillation	8.50%
Himmelreich *et al.* [16]	Ischemic stroke	379	Holter monitoring	13 day	Atrial fibrillation	2.64%
Lyckhage *et al.* [17]	Ischemic stroke	39641	cECG	120 day	Atrial fibrillation	6.30%

**Abbreviations:** cECG: continuous electrocardiography; SVEB: supraventricular extrasystole; VEB: ventricular extrasystole; SVRs: short supraventricular runs; NSVT: nonsustained ventricular tachycardia.

## LIT OF ABBREVIATIONS

AF: Atrial Fibrillation
BBB: Blood-brain Barrier
cAMP: Cyclic Adenosine Monophosphate
CAN: Centralized Autonomic Nervous
ECG: Electrocardiogram
HRV: Heart Rate Variability
PAG: Periaqueductal Gray
ROS: Reactive Oxygen Species
SHS: Stroke-heart Syndrome
